# Sequential Treatment With Corticosteroids and Cyclosporine A in a High-Risk Patient With IgG-Negative Immunotactoid Glomerulopathy

**DOI:** 10.7759/cureus.104280

**Published:** 2026-02-26

**Authors:** Masatoshi Inoue, Chikara Asai, Naoki Kamegai, Junichiro Yamamoto

**Affiliations:** 1 Nephrology, Nagoya University Graduate School of Medicine, Nagoya, JPN; 2 Nephrology, Asahi University Hospital, Gifu, JPN

**Keywords:** cyclosporine a, electron microscopy, fibrillary glomerulonephritis, immunotactoid glomerulopathy, nephrotic syndrome

## Abstract

Immunotactoid glomerulopathy (ITG) is a rare glomerular disease characterized by organized microtubular deposits and is frequently associated with hematologic malignancies. Diagnosis is typically supported by IgG-positive staining on immunofluorescence together with characteristic ultrastructural findings on electron microscopy. However, diagnostic difficulties may arise when immunofluorescence findings are atypical. We report the case of an 82-year-old woman who presented with nephrotic syndrome and progressive renal dysfunction, with proteinuria of 13.8 g/g creatinine, a serum creatinine level of 2.33 mg/dL, and an estimated glomerular filtration rate of 16.0 mL/min/1.73 m². Serum and urine immunoelectrophoresis revealed no evidence of monoclonal proteins. Renal biopsy demonstrated mesangial proliferative glomerulonephritis with negative IgG staining on immunofluorescence. The diagnosis of ITG was ultimately established by electron microscopy, which revealed organized hollow microtubular structures measuring 30 to 90 nm in diameter within the mesangial and subepithelial regions. Based on the presence of hypertension, nephrotic-range proteinuria, and impaired baseline renal function, the patient was considered to be at high risk for progression to end-stage renal disease. No underlying hematologic malignancy was identified. Treatment with corticosteroids followed by cyclosporine A (CsA) resulted in a favorable clinical response. By day 364, the patient achieved near-complete remission with proteinuria decreasing to 0.24 g/g creatinine and eGFR stabilizing at 25.4 mL/min/1.73 m². This case underscores the importance of considering ITG when characteristic ultrastructural findings are present, even in the absence of IgG positivity on immunofluorescence. Furthermore, this case may represent one of the first reported instances in which treatment with prednisolone and CsA was effective in a high-risk patient with ITG without an identifiable clonal disorder.

## Introduction

Immunotactoid glomerulopathy (ITG) is a rare glomerular disease characterized by organized microtubular deposits composed predominantly of immunoglobulins. It accounts for about 0.06% of native renal biopsies and is considered a distinct entity from fibrillary glomerulonephritis (FGN), in which randomly arranged fibrils are seen instead of microtubules [1]. Electron microscopy is essential for diagnosis because the microtubules in ITG are typically larger (usually 30-90 nm in diameter) and display an ordered parallel or stacked arrangement [2].

Clinically, ITG commonly presents with nephrotic-range proteinuria, hematuria, and progressive renal dysfunction. A remarkable feature of ITG is its strong association with underlying B-cell lymphoproliferative disorders or monoclonal gammopathies, which have been reported in approximately 66% of cases [3]. Therefore, a detailed hematologic evaluation is recommended in all suspected patients. However, a subset of patients shows no detectable hematologic disease despite exhaustive evaluation, and the optimal management strategy for such cases remains uncertain. While clone-directed therapy is advocated when a hematologic disorder is identified [4], immunosuppressive therapy is sometimes used in patients without detectable clonal abnormalities, although the efficacy of this approach is still debated.

While IgG-positive staining on immunofluorescence is a hallmark of ITG, atypical cases with negative staining can present significant diagnostic challenges, as they may mimic other glomerulopathies. Here, we report a case of IgG-negative ITG without any evidence of underlying hematologic malignancy, in which the diagnosis was established primarily by renal biopsy and electron microscopy and successfully treated with prednisolone (PSL) and cyclosporine A (CsA). This case highlights the importance of ultrastructural analysis in the face of atypical immunohistochemical findings, as well as the therapeutic considerations in ITG without associated hematologic disease.

## Case presentation

An 82-year-old woman presented to her primary physician with progressive dyspnea on exertion and a rapid seven kg weight gain over one month and was subsequently referred to our hospital. The patient had no history of recent viral infections, pre-existing hypertension, or other preceding illnesses. There were no changes in her regular medications, and no specific triggers for the onset of symptoms were identified. The patient's serum creatinine level was confirmed to have been within the normal range during her most recent health check-up prior to the current presentation. On admission, her blood pressure was 167/88 mmHg, heart rate 86 beats/min, and oxygen saturation 96% on room air. Her height was 145.7 cm and body weight 67.7 kg, and grade 3+ bilateral pitting edema of the lower extremities extending to the knees was noted, although no periorbital edema was present. Laboratory evaluation revealed nephrotic syndrome with a serum albumin level of 2.7 g/dL and urine protein excretion of 13.8 g/g creatinine (Table 1). Renal function was impaired, with a serum creatinine of 2.33 mg/dL and an estimated glomerular filtration rate (eGFR) of 16.0 mL/min/1.73 m². Serum and urine immunoelectrophoresis demonstrated no monoclonal proteins.

**Table 1:** Laboratory investigations of this patient. eGFR: estimated glomerular filtration rate; PR3-ANCA: proteinase 3-antineutrophil cytoplasmic antibodies; MPO-ANCA: myeloperoxidase-antineutrophil cytoplasmic antibodies; HPF: high-power field; ND: not done.

Variable	Reference Range	On Admission	Day 21	Day 364
Hemoglobin (g/dL)	11.6-14.8	9.3	8.4	10.3
Hematocrit (%)	35.1-44.4	28.1	25	29.9
White blood cell count (per μL)	3000-8600	5870	7310	6800
Platelet count (per μL)	158,000-348,000	183,000	115,000	126,000
Creatinine (mg/dL)	0.46-0.79	2.33	1.53	1.92
Urea nitrogen (mg/dL)	8-22	31.9	45.3	54.4
eGFR (mL/min/1.73m²)	>60	16.0	19.8	25.4
Albumin (g/dL)	4-5	2.7	2.3	3.0
Sodium (mmol/L)	138-146	144	144	135
Potassium (mmol/L)	3.6-4.9	4.4	3.6	3.6
Chloride (mmol/L)	99-109	109	107	96
Calcium (mg/dL)	8.6-10.2	8.5	8.2	9.2
Phosphorus (inorganic) (mg/dL)	2.5-4.5	4.5	ND	3.6
Lactate dehydrogenase (IU/L)	124-222	320	ND	315
Total cholesterol (mg/dL)	65-163	393.9	ND	151
Triglyceride (mg/dL)	30-117	217	ND	183
IgG (mg/dL)	700-1600	518	ND	ND
IgA (mg/dL)	100-490	147	ND	ND
IgM (mg/dL)	50-320	37	ND	ND
C3 (mg/dL)	50-120	92	ND	ND
C4 (mg/dL)	13-54	26	ND	ND
Antinuclear antibody	<40	<40	ND	ND
Cryoglobulin	Negative	Negative	ND	ND
PR3-ANCA (U/mL)	Negative	Negative	ND	ND
MPO-ANCA (U/mL)	Negative	Negative	ND	ND
Urine protein (g/g creatinine)	<0.1	13.8	3.8	0.24
Urine red blood cells (/HPF)	<0.1	50-99	20-29	0-9

**Figure 1:**
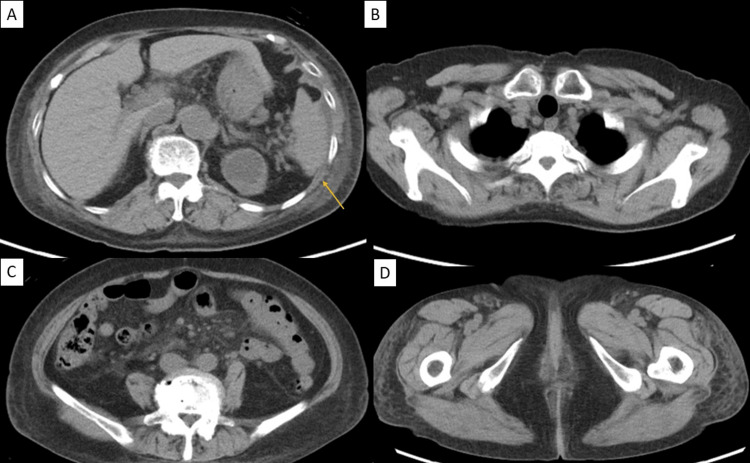
Computed tomography findings on admission. Whole-body computed tomography was performed to screen for underlying hematologic malignancies. (A) Abdominal CT showing a normal-sized spleen (arrow) without evidence of splenomegaly. (B) Chest, (C) abdominal, and (D) pelvic CT images demonstrating no significant lymphadenopathy in the cervical, axillary, mesenteric, or inguinal regions.

Whole-body computed tomography showed no lymphadenopathy or splenomegaly (Figure 1).

**Figure 2:**
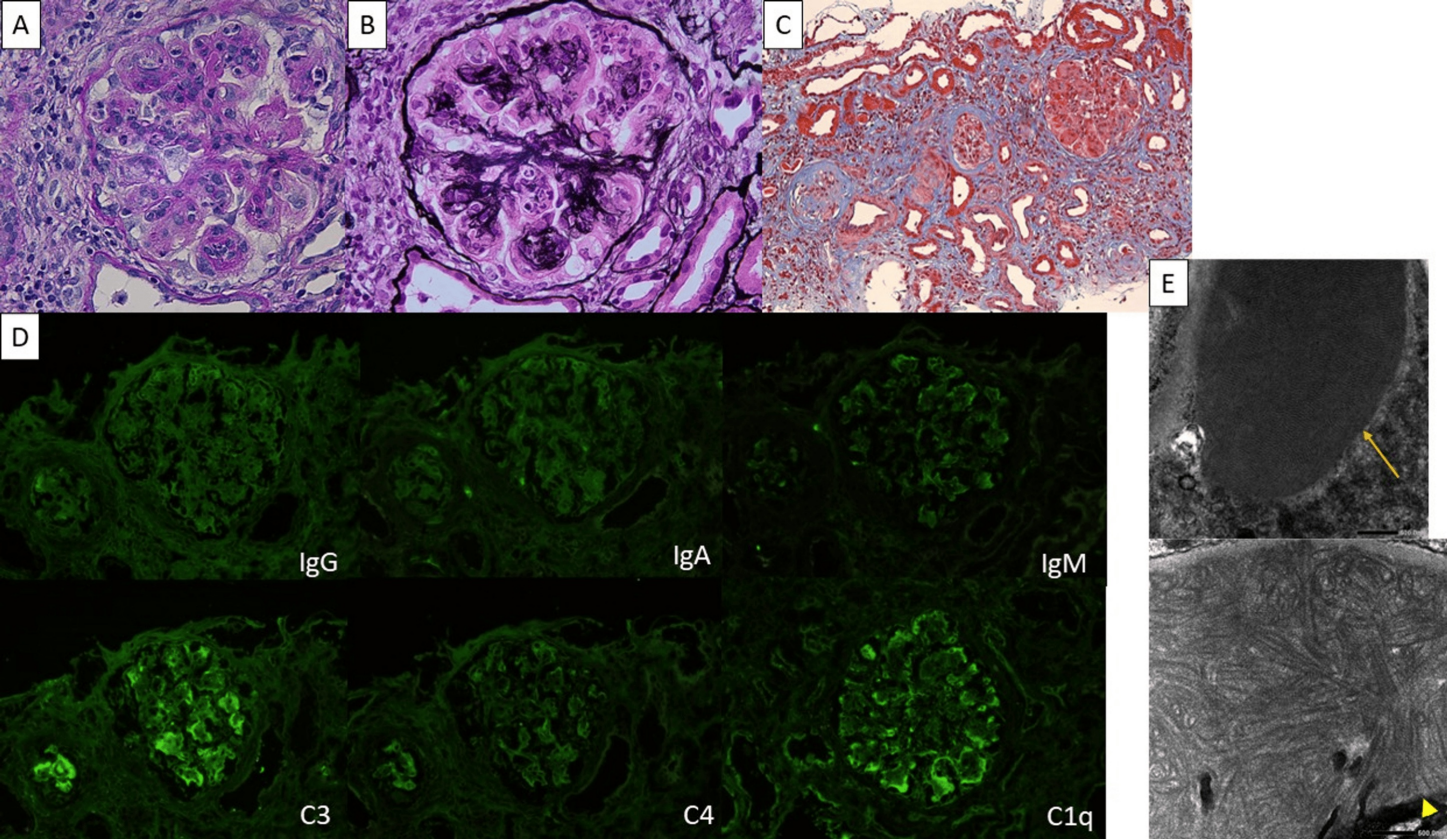
Pathological findings of the renal biopsy. (A) Periodic acid–Schiff (PAS) stain (original magnification ×400) and (B) periodic acid–methenamine silver (PAM) stain (original magnification ×400) showing mesangial matrix expansion and mesangial cell proliferation. (C) Masson’s trichrome stain demonstrating tubular atrophy and interstitial fibrosis, indicating chronic parenchymal damage. (D) Immunofluorescence microscopy showing negative staining for IgG, IgA, and IgM. C3 shows positive staining, whereas C4 is negative, and C1q is trace to weakly positive. (E) Electron microscopy showing organized hollow microtubular and cylindrical deposits measuring 30–90 nm in diameter. Arrows indicate deposits predominantly located within the mesangial area, whereas arrowheads indicate deposits located within and along the glomerular basement membrane. The microtubules are arranged in interweaving bundles, a characteristic ultrastructural feature of immunotactoid glomerulopathy. Scale bars represent 500 nm.

Serological testing was negative for antinuclear antibodies and cryoglobulins. She was hospitalized with a diagnosis of nephrotic syndrome, and a renal biopsy was performed on hospital day 2. On the same day, intravenous methylprednisolone pulse therapy (500 mg/day for 3 days) was initiated, followed by oral PSL at 40 mg/day. Following these treatments, her proteinuria decreased gradually, reaching 7.84 g/gCr on day 8. Renal biopsy revealed mesangial matrix expansion and mesangial cell proliferation on light microscopy (Figure 2). Electron microscopy revealed organized hollow microtubular structures measuring approximately 30-90 nm in diameter. These deposits were located predominantly within the mesangium and in the subepithelial space along the glomerular basement membranes, appearing as interweaving bundles of distinct microtubules.

Based on these ultrastructural findings, a diagnosis of ITG was made. Although her proteinuria decreased to 3.8 g/g creatinine, CsA (50 mg/day) was added on day 22, and PSL was gradually tapered. She was discharged on hospital day 30.

During outpatient follow-up, PSL was continuously tapered and her proteinuria gradually decreased. At one year after diagnosis, urinary protein excretion had decreased to 0.2 g/g creatinine, serum creatinine was 1.92 mg/dL with an eGFR of 19.8 mL/min/1.73 m², and serum albumin had improved to 3.0 g/dL, indicating a favorable clinical course with sustained renal function and near-complete remission of proteinuria.

## Discussion

In our patient, corticosteroid therapy led to an initial reduction in proteinuria, but significant protein loss persisted. CsA was subsequently introduced. While the therapeutic response emerged gradually, complete remission was eventually achieved. Although these agents were administered sequentially, making it challenging to isolate their individual contributions, the combined use of corticosteroids and CsA from the early phase of treatment has not been previously reported in ITG.

Differential diagnosis of glomerular diseases characterized by organized deposits on electron microscopy includes systemic lupus erythematosus, cryoglobulinemic glomerulonephritis, amyloidosis, FGN, and ITG. In our case, systemic diseases such as lupus nephritis and cryoglobulinemic glomerulonephritis were ruled out based on negative serological findings, and amyloidosis was excluded due to the absence of Congo red staining. Cryoglobulinemic glomerulonephritis can also present with similar microtubular structures, but these fibrils are typically smaller in diameter (25-35 nm) than those in ITG. Furthermore, while cryoglobulinemic deposits rarely involve the subepithelial space, ITG frequently exhibits subepithelial deposits, which were observed in the present case [5]. Distinguishing ITG from FGN is also crucial, as the two entities share similar clinical presentations but differ in ultrastructural characteristics. FGN is characterized by randomly arranged, solid fibrils that are typically smaller (10-30 nm) than the microtubules seen in ITG [1]. Regarding immunohistochemical markers, DNAJB9 has recently emerged as a highly sensitive and specific marker for FGN [2], but its availability remains limited, and this staining could not be performed at our institution. Therefore, the diagnosis of ITG in this case was established based on the presence of organized hollow microtubules with a larger diameter (30-90 nm). Historically, IgG-positive staining on immunofluorescence has been considered a hallmark of ITG. Although immunofluorescence was negative for IgG in the present case, it has been reported that certain antibody clones may fail to detect the deposits. In such cases, re-staining with alternative antibody clones is recommended [6]. Unfortunately, since the biopsy specimens had already been discarded, additional staining could not be performed. Consequently, it remained unclear whether the ITG in this patient was monoclonal or polyclonal in nature.

This distinction regarding the clonal nature of ITG is clinically significant because prognosis can vary substantially. Progression to end-stage renal disease (ESRD) has been reported to be more frequent in patients with polyclonal ITG compared to those with monoclonal disease, at 53% versus 11% [3]. Furthermore, regardless of the clonal type, it is well established that certain clinical features, such as hypertension, nephrotic-range proteinuria, chronic kidney disease, and extensive glomerular involvement, indicate a poor prognosis [7]. Notably, our patient fulfilled all of these adverse prognostic criteria on presentation. Given the presence of these combined factors, there was an exceptionally high risk of progression to ESRD in this case.

When an underlying pathogenic driver is identified, the range of treatment options broadens. For instance, in ITG cases complicated by monoclonal immunoglobulin deposition disease, bortezomib-based regimens have shown efficacy [4]. In contrast, therapeutic decision-making is considerably more difficult in patients like ours, in whom no hematologic abnormalities are detected. While rituximab has been increasingly utilized in recent years, its benefits remain inconsistent. Some reports have noted relapses and refractory disease [8], and others have even shown recurrence following kidney transplantation despite prior B-cell depletion with rituximab [9].

CsA is a calcineurin inhibitor traditionally employed for various nephrotic glomerular diseases. Interestingly, it has also been shown to reduce proteinuria in Alport syndrome, which is a hereditary basement membrane disorder that does not involve immune-mediated mechanisms [10]. Recent experimental studies using adriamycin-induced nephropathy models demonstrated that CsA can restore foot process architecture and modulate cytoskeletal regulators, including the actin-cross-linking protein transgelin, through the upregulation of Krüppel-like factor 4. It also preserves nephrin and synaptopodin expression [11]. These findings suggest that CsA may exert podocyte-stabilizing effects through multiple pathways.

## Conclusions

In conclusion, this case demonstrates that ITG can present with atypical immunofluorescence findings, specifically the absence of IgG staining, making electron microscopy indispensable for a definitive diagnosis. Furthermore, this case demonstrates that a combination of corticosteroids and CsA may be an effective and viable therapeutic option for high-risk ITG patients who lack identifiable hematologic malignancies or clonal disorders. Beyond systemic immunosuppression, the clinical response in this case may be attributed to the podocyte-stabilizing effects of CsA, which could help stabilize renal function and achieve remission in patients with poor prognostic features. Further studies are required to establish a standardized treatment protocol and to further elucidate the underlying mechanisms of this therapeutic approach.
